# Nintedanib overcomes drug resistance from upregulation of FGFR signalling and imatinib‐induced KIT mutations in gastrointestinal stromal tumours

**DOI:** 10.1002/1878-0261.13199

**Published:** 2022-03-06

**Authors:** Juan Liu, Jingjing Gao, Aoli Wang, Zongru Jiang, Shuang Qi, Ziping Qi, Feiyang Liu, Kailin Yu, Jiangyan Cao, Cheng Chen, Chen Hu, Hong Wu, Li Wang, Wenchao Wang, Qingsong Liu, Jing Liu

**Affiliations:** ^1^ Anhui Province Key Laboratory of Medical Physics and Technology, CAS Key Laboratory of High Magnetic Field and Ion Beam Physical Biology Institute of Health and Medical Technology Hefei Institutes of Physical Science Chinese Academy of Sciences Hefei China; ^2^ University of Science and Technology of China Hefei China; ^3^ Hefei Cancer Hospital Chinese Academy of Sciences Hefei China; ^4^ Precision Medicine Research Laboratory of Anhui Province Hefei China

**Keywords:** FGFR, GISTs, imatinib resistance, KIT, nintedanib

## Abstract

Drug resistance remains a major challenge in the clinical treatment of gastrointestinal stromal tumours (GISTs). While acquired on‐target mutations of mast/stem cell growth factor receptor (KIT) kinase is the major resistance mechanism, activation of alternative signalling pathways may also play a role. Although several second‐ and third‐generation KIT kinase inhibitors have been developed that could overcome some of the KIT mutations conferring resistance, the low clinical responses and narrow safety window have limited their broad application. The present study revealed that nintedanib not only overcame resistance induced by a panel of KIT primary and secondary mutations, but also overcame ERK‐reactivation‐mediated resistance caused by the upregulation of fibroblast growth factor (FGF) activity. In preclinical models of GISTs, nintedanib significantly inhibited the proliferation of imatinib‐resistant cells, including GIST‐5R, GIST‐T1/T670I and GIST patient‐derived primary cells. In addition, it also exhibited dose‐dependent inhibition of ERK phosphorylation upon FGF ligand stimulation. *In vivo* antitumour activity was also observed in several xenograft GIST models. Considering the well‐documented safety and pharmacokinetic profiles of nintedanib, this finding provides evidence for the repurposing of nintedanib as a new therapy for the treatment of GIST patients with *de novo* or acquired resistance to imatinib.

## Graphical abstract

Drug resistance remains a major challenge in the clinical treatment of gastrointestinal stromal tumors (GISTs). Here, we observed that nintedanib can overcome imatinib resistance induced by cKIT secondary mutations and elevated FGF ligand expression, thus providing a new potential therapy for patients with GIST that acquired resistance to Imatinib.

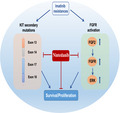

**Abbreviations**
FGFs: fibroblast growth factors
FRS2: FGFR substrate 2
GIST: gastrointestinal stromal tumours
H2AX: histone 2A family member X
IPF: idiopathic pulmonary fibrosis
KIT: mast/stem cell growth factor receptor
MAPK: mitogen‐activated protein kinase
NSCLC: non‐small‐ cell lung cancer
PI3K: phosphoinositide 3‐kinase
RTK: receptor tyrosine kinase

## 1 Introduction

Gastrointestinal stromal tumours (GISTs) are mesenchymal tumours that usually occur in the gastrointestinal tract [1]. Approximately 15 000 new GIST patients are diagnosed annually worldwide [2, 3]. Nearly 85% of GISTs bear oncogenic mutations in mast/stem cell growth factor receptor (KIT), a member of the class III receptor tyrosine kinase (RTK) family, which results in persistent receptor‐initiated signals and subsequently activates downstream effectors [4, 5]. Substitution mutations, such as V559A/D/G and L576P, are located in the juxtamembrane domain which inserts into the active site of the kinase and suppresses the formation of the active structure [6]. Secondary drug‐resistant mutations, including T670I and V654A, are located at the ATP‐binding pocket, while D816E/H/V, D820E/G/Y and N822K mutations are in activation loops.

The discovery of imatinib (Glivec), a targeted tyrosine kinase inhibitor, has led to dramatic changes in the clinical management of GISTs [7]. Approximately 80%–90% of GIST patients initially achieve disease stabilization after imatinib treatment, which is typically indicated for unresectable or disseminated disease [8]. However, approximately 50% of patients with GIST develop secondary resistance within 2–3 years of treatment with imatinib [9]. A variety of factors lead to the emergence of imatinib resistance, with the most common being secondary drug‐resistance‐conferring mutations in the KIT kinase domain, including the T670I ‘gatekeeper’ mutation [10, 11]. Regorafenib and sunitinib were approved for imatinib‐resistant GISTs with primary and secondary mutations [12, 13]. However, the clinical benefits of these treatments are limited by toxicity and poor overall response rates [14, 15]. Avapritinib and ripretinib were approved in 2020 for GIST patients harbouring PDGFRA exon 18 mutation and advanced GIST who have received prior treatment with three or more kinase inhibitors, including imatinib, respectively [16]. While side effects were seen with these drugs, including intracranial haemorrhage, severe headache and risk in secondary tumour induction [17, 18]. Although ponatinib and pazopanib are in clinical trials and could also overcome the T670I mutation‐related resistance to imatinib, severe side effects, such as a high risk of hypertension and arterial occlusive disease, might greatly affect the patient response to these drugs [19, 20, 21, 22]. Therefore, the need for safe and effective drugs that can overcome imatinib resistance remains critical.

Fibroblast growth factors and receptors (FGFR1–4) are involved in the regulation of cell survival and proliferation and angiogenesis [23]. FGF/FGFRs can regulate several downstream intracellular pathways through the intracellular receptor substrate FGFR substrate 2 alpha (FRS2α), resulting in subsequent upregulation of the RAS/mitogen‐activated protein kinase (MAPK) and phosphoinositide 3‐kinase (PI3K)/AKT signalling pathways [24, 25]. It has been shown that reactivation of MAPK signalling mediated by FGFR could reduce imatinib sensitivity after long‐term imatinib treatment [26]. The simultaneous inhibition of the KIT and FGFR signalling pathways may be beneficial for some patients with GISTs in overcoming resistance to targeted therapies. Hence, development of additional targeted inhibitors that can overcome both KIT secondary mutations and FGFR feedback activation remains critical.

Through high‐throughput screening with functional isogenic BaF3 cells, we found that nintedanib (BIBF 1120), an inhibitor of three angiokinases, VEGFR/PDGFR/FGFR [27] that has been approved for the treatment of idiopathic pulmonary fibrosis (IPF) [28] and non‐small‐cell lung cancer (NSCLC) [29], showed strong inhibition against the effects of multiple mutations in KIT kinase, especially the T670I mutation. In addition, nintedanib also inhibited ERK phosphorylation upon FGF ligand stimulation in a dose‐dependent manner.

## 2 Materials and methods

### 2.1 Chemicals

Nintedanib (BIBF 1120), sunitinib, imatinib, avapritinib (BLU‐285) and ripretinib (DCC‐2618) were obtained from MedChemExpress (Shanghai, China).

### 2.2 Antibodies and immunoblotting

The following antibodies were purchased from Cell Signaling Technology (Danvers, MA, USA): Phospho‐KIT (Tyr703; D12E12) rabbit mAb (no. 3073), Phospho‐KIT (Tyr719) antibody (no. 3391), Phospho‐anti‐KIT (pY823) rabbit mAb (no. 77522), KIT (Ab81) mouse mAb (no. 3308), Phospho‐AKT (Thr308; 244F9) rabbit mAb (no. 4056), Phospho‐AKT (Ser473; D9E) XP rabbit mAb (no. 4060), AKT (pan; C67E7) rabbit mAb (no. 4691), Phosphop44/42 MAPK (ERK1/2; Thr202/Tyr204; 197G2) rabbit mAb (no. 4377), p44/42 MAPK(ERK1/2; 137F5) rabbit mAb (no. 4695), PhosphoSTAT3 (Tyr705; D3A7) XP rabbit mAb (Biotinylated; no. 4093), STAT3 (D3Z2G) rabbit mAb (no. 12640), GAPDH (D16H11) XP rabbit mAb (no. 5174), Phospho‐FRS2 (Tyr196) antibody (no. 3864), Phospho‐Histone H2A.X (Ser139; 20E3) rabbit mAb (no. 9718), PARP (46D11) rabbit mAb (no. 9532), and caspase‐3 (8G10) rabbit mAb (no. 9665). β‐actin antibody was purchased from TransGen Biotech (Beijing, China; no. HC201‐02).

### 2.3 BaF3 isogenic cell lines generation

Functional BaF3 cell lines were generated as described previously [30]. Briefly, the *KIT* wt and mutant genes were cloned into the pMSCVpuro retroviral vector for virus production in combination with two helper plasmids in HEK‐293T cells. Virus‐containing supernatant was used to infect BaF3 cells followed by puromycin selection and IL‐3 withdrawal to obtain the stable *KIT*‐overexpressing BaF3 cells. These functional BaF3 cell lines were independent of IL‐3 for cell survival and proliferation.

### 2.4 Cell lines and cell culture

GIST‐T1‐T670I cell line was generated using CRISPR/Cas9 system as described previously [31]. GIST‐T1 cell line was purchased from Cosmo Bio Co., Ltd, Tokyo, Japan. GIST‐882 and GIST‐48B were a gift from J. A. Fletcher (Brigham and Women’s Hospital in Boston, USA). GIST‐5R cell line was a gift from B. Rubin (Lerner Research Institute, USA). GIST‐5R, GIST‐T1‐T670I and GIST‐T1 cell lines were cultured in Dulbecco's Modified Eagle Medium (DMEM; Corning, Manassas, VA, USA) with 10% FBS (ExCell Bio, Guangzhou, China). GIST‐882 and GIST‐48B cell lines were cultured in IMDM (Gibco by Life Technologies, Carlsbad, CA, USA) with 10% FBS (GIBCO). BaF3 isogenic cell lines were cultured in RPMI1640 with 10% FBS. Primary cells were cultured in DMEM/F12 with 5% FBS (GIBCO), Glutamax‐I (GIBCO), primocin (InvivoGen, San Diego, CA, USA), 5 μg·mL^−1^ insulin (GIBCO), 25 μg·mL^−1^ hydrocortisone (Sigma, St. Louis, MO, USA), 125 ng·mL^−1^ EGF (Sigma) and 10 μm Rho kinase inhibitor y27632 (Haoyuan Chemexpress Inc, Shanghai, China) [30].

### 2.5 Cell proliferation assay

The method has been described previously [30]. Briefly, the GISTs cell lines and human primary GIST cells were cultured in 96‐well plates. Each well was cultured in a volume of 100 μL at a density of about 3000 cells. They were treated with drugs (0–10 μm) for 3 or 6 days with or without 20 ng·mL^−1^ FGF2 after overnight culturing. CellTiter Glo reagent (Promega, Madison, WI, USA) is used to measure cell viability and it is calculated by graphpad prism 8.0.1 (Graphpad, San Diego, CA, USA).

### 2.6 Biochemical assay of kinase activity

The biochemical assay of a series of KIT protein was tested by Invitrogen (Carlsbad, CA, USA) as described previously [30]. Briefly, the KIT wt and mutant proteins (5–20 ng·μL^−1^) were treated with serially diluted nintedanib (0.5–10 000 nm), and substrate Poly (4 : 1 Glu, Tyr) peptide (0.4 μg·μL^−1^) with 20 μm ATP. The reaction in each tube was started immediately by adding ATP and kept going for an hour at room temperature. Then the ADP‐Glo reagent was added into each well to stop the reaction and consume the remaining ATP within 40 min. At the end, kinase detection reagent was added into the well and incubated for 30 min to produce a luminescence signal. Luminescence signal was measured with an automated plate reader and the dose–response curve was fitted using prism 5.0 (GraphPad Software).

### 2.7 Western blot analysis

GIST‐T1, GIST‐882, GIST‐5R and GIST‐48B cells were treated with DMSO, serially diluted nintedanib, 1 μm imatinib and 1 μm sunitinib for 4 h. Patient‐derived primary cells were also treated with various concentration of drugs for 4 h with or without FGF2 (20 ng·mL^−1^). Immunoblot analysis was done as described previously [32]. Briefly, the cells were washed with cold PBS and lysed in RIPA buffer (Beyotime, Shanghai, China) with a protease inhibitor cocktail (CST). The protein lysates after sonication and boiling were separated by electrophoresis using 10% SDS/PAGE and transferred to a NC membrane (Millipore, Bedford, MA, USA). After being blocked with 1 × TBS containing 0.1% Tween‐20 and 5% non‐fat milk, the membrane was then incubated with antibodies. Finally, bound secondary antibody was visualized using ECL Western Blotting Detection Kit (Millipore).

### 2.8 Cell cycle analysis

The assay was tested by flow cytometry (BD, San Jose, CA, USA) as published previously [30, 33]. Briefly, the GISTs cells were cultured in 12‐well plates overnight, then treated with nintedanib (0.03–1 μm), sunitinib and imatinib. The cells were fixed in 70% cold ethanol, then stained using PI/RNase staining buffer (BD). Finally, a FACS Calibur flow cytometer (BD Biosciences) was used to test the cell cycle distribution. The results were analysed by modifit software (Verity Software House, West Lafayette, IND, USA).

### 2.9 Apoptosis detection

GIST‐T1, GIST‐882, GIST‐5R and GIST‐48B cells were treated with DMSO, serial dilutions of nintedanib (0.03–1 μm), 1 μm imatinib or 1 μm sunitinib for 24 h and 72 h. The cells were then collected and analysed by western blotting using PARP, β‐actin and caspase3 antibody as described previously [30, 33].

### 2.10 Mouse xenograft study

BALB/C‐nu mice (6‐week female) were obtained from Nanjing Biomedical Research Institute and maintained as described previously [34]. Briefly, the animals were housed in an air‐conditioned animal room at a temperature of 24 ± 2 °C and a relative humidity of 50 ± 10% in a specific pathogen‐free facility. The animals were allowed free access to water and laboratory diet. All studies were approved by the Hefei Institutes of Physical Science ethics committee, Chinese Academy of Sciences (approval number: DWLL‐2018‐016). Imatinib, nintedanib and sunitinib were dissolved in 0.5% Methylcellulose and 0.4% Tween 80 solution. Tumours were established by subcutaneous implantation of one million cells (BaF3‐KIT‐T670I) or five million cells (GIST‐T1, GIST‐5R and GIST‐T1‐T670I) or ten million (GIST882) into the right flank of BALB/C‐nu mice. After tumour cell inoculation, animal body weights and tumour volumes were measured. When tumour volumes reached 200–300mm^3^, mice were divided into groups randomly and treated with drugs. Imatinib, nintedanib and sunitinib were delivered daily by orally gavage. At a predefined time point, animal body weights and tumour volumes were measured, and tumour volume was calculated (width^2 ^× length/2). When the tumour size of xenografts reached ~ 2000 mm^3^, biopsies were obtained and the mice were castrated. The tumour growth inhibition (TGI) was calculated according to actual tumour weight using the formula: (*W*_vehicle_ − *W*_Test_)/*W*_Vehicle_ × 100% in which *W* is defined as actual tumour weight. Standard procedures of immunohistochemistry staining have been described previously [30]. Briefly, tumour tissue samples were processed for paraffin embedding. The sections were stained with haematoxylin and eosin (H&E), human Ki‐67 (ZSGB‐BIO, Beijing, China) and *in situ* Cell Death Detection Kit (POD; Roche, Mannheim, Germany), respectively.

## 3 Results

### 3.1 Nintedanib exhibits potent effects against a panel of primary and secondary KIT kinase mutations

**Fig. 1:**
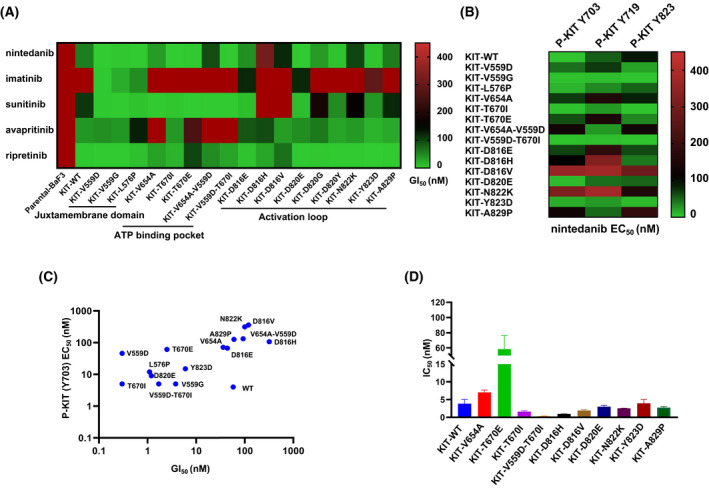
Nintedanib inhibits KIT wt and KIT mutant *in vitro*. (A) The heatmap shows GI_50_ values (growth inhibitory activity) of nintedanib, imatinib, sunitinib, avapritinib and ripretinib in BaF3 isogenic cell lines whose proliferation were depend on KIT wt and KIT mutant kinase. The cell lines were treated with drugs (0–10 μm) for 3 days (*n* = 3, independent experiments). (B) The heatmap shows that EC_50_ (effective concentrations) values was calculated by quantifying the levels of KIT Y703/Y719/Y823 relative to KIT after a various dose of nintedanib treatment for 2 h. (C) Relativity between GI_50s_ and EC_50s_ on KIT wt and mutants of BaF3 isogenic cell lines panel. (D) Nintedanib inhibits KIT wt and KIT mutant proteins using ADP‐Glo biochemical assay. Data are shown as mean ± SD (*n* = 2, independent experiments). High GI_50_ (EC_50_) and low GI_50_ (EC_50_) values were corresponded by red colour and green colour, respectively.

We first tested the antiproliferative effects of nintedanib, sunitinib, imatinib, avapritinib and ripretinib against a panel of BaF3 cells transformed by KIT kinase; their proliferation depends on the KIT kinase (Fig. 1A, Table S1). The results demonstrated that nintedanib displayed better potency than sunitinib or imatinib in KIT wt BaF3 cells (GI_50_ values were 0.058, 0.59 and 0.11 μm, respectively). For primary mutations in the juxtamembrane domain, including L576P and V559D/G, nintedanib exhibited better activity than sunitinib, imatinib, avapritinib and ripretinib. Nintedanib, sunitinib and ripretinib were highly effective in BaF3 cells with the KIT V654A mutation and the mixed V654A/V559D and T670I/V559D mutations, which are secondary imatinib‐resistant mutations in the ATP‐binding pocket. Interestingly, for BaF3 cells with the imatinib‐resistant gatekeeper mutation T670I, nintedanib was more potent than sunitinib, avapritinib and ripretinib (GI_50_ < 0.0003 μm vs 0.005 μm; 0.039μm; 0.017μm). In addition, nintedanib showed similar potencies to avapritinib and ripretinib, and the efficacy is better than sunitinib or imatinib in activating KIT loop mutations, such as D816H/V and D820E/G/Y. Overall, these results suggested that most of the secondary imatinib‐resistant mutations in the activation loop and ATP‐binding pocket of KIT are sensitive to nintedanib, especially the gatekeeper mutation T670I, compared with sunitinib and imatinib, avapritinib and ripretinib.

To ensure that the antiproliferative effects of nintedanib in engineered BaF3 cells were due to mutations in on the KIT signalling pathway, we then examined its inhibitory effects on KIT Y703, Y719 and Y823 phosphorylation (Fig. 1B, Fig. S1 and Table S2). Western blotting showed that nintedanib potently inhibited the autophosphorylation of KIT wt and KIT with V559A/D/G, L576P, T670I, D820E, Y823D or A829P mutations but was much less potent on the autophosphorylation of KIT with D816V and N822K mutations, which showed similar trends to the growth inhibition of the KIT BaF3 cells (Fig. 1C). In addition, to test the inhibitory activity of nintedanib in cells with KIT wt and mutant proteins, we used an ADP‐Glo biochemical assay (Fig. 1D, Table S3). The data revealed that nintedanib was potent against KIT wt (IC_50_ = 3.85 nm), KIT V654A (IC_50_ = 7.94 nm), KIT T670E (IC_50_ = 59.4 nm), KIT T670I (IC_50_ = 1.65 nm), KIT D816H and D816V (IC_50_ = 1.14 nm and 2.38 nm, respectively), KIT D820E (IC_50_ = 3.14 nm), KIT N822K (IC_50_ = 2.6 nm), KIT Y823D (IC_50_ = 3.5 nm), and KIT A829P (IC_50_ = 2.64 nm). It was more potent against the mixed mutation T670I/V559D (IC_50_ < 0.5 nm) but relatively less potent against the KIT T670E mutation. These data suggested that nintedanib can suppress the proliferation of a panel of BaF3 cells due to its inhibitory effect on primary and secondary KIT kinase mutations.

### 3.2 Nintedanib inhibits the proliferation of GIST cell lines and human primary GIST cells through the KIT signalling pathway

**Fig. 2:**
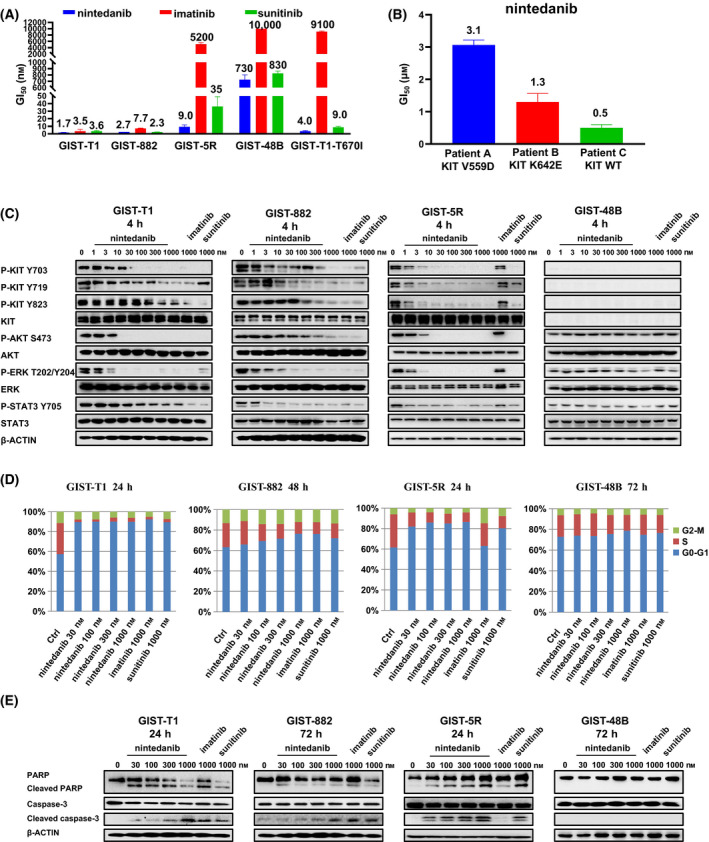
Effects of nintedanib on human GIST cell lines and human primary GIST cells. (A) Antiproliferative effects of nintedanib against GIST‐T1, GIST‐882, GIST‐5R, GIST‐48B and GIST‐T1‐T670I cell lines. The cell lines were treated with drugs (0–10 μm) for 3 days using CellTiter‐Glo assay. Data are shown as mean ± SD (*n* = 3, independent experiments). (B) Anti‐proliferation of human primary GIST cells after treatment with nintedanib for 6 days using CellTiter‐Glo assay. Data are shown as mean ± SD (*n* = 3, independent experiments). (C) Inhibition of signalling pathways of KIT in GIST‐T1, GIST‐882, GIST‐5R and GIST‐48B cell lines after treatment with nintedanib for 4 h (immunoblotting; *n* = 3, independent experiments). (D) Effects of nintedanib on cell cycle progression after treatment for 24–72 h (flow cytometry). This experiment was carried out once. (E) Nintedanib induced GIST‐T1, GIST‐882, GIST‐5R and GIST‐48B cell line apoptosis. This experiment was conducted once.

We next assessed the antiproliferative effects of nintedanib against a series of GIST cell lines and human primary GIST cells derived from three GIST patients expressing KIT wt, KIT‐V559D or KIT‐K642E mutations. The study showed that compared with imatinib and sunitinib, nintedanib was more potent against GIST‐T1 cells (GI_50_ = 1.7 nm; Fig. 2A), which harbour the Δ560–578 mutation in the juxtamembrane region of KIT. Nintedanib also showed potency similar to sunitinib against GIST882 cells, which harbours the primary K642E mutation in the KIT c‐helix, but it was more potent than imatinib. In addition, nintedanib was more effective than sunitinib in the GIST‐T1‐T670I and GIST‐5R cell lines, which are both imatinib resistant and harbour gatekeeper T670I and Δ560–578 KIT mutations. For GIST‐48B cells, which are KIT‐independent cells, none of the three compounds showed activity. Similarly, in human primary GIST cells harbouring KIT wt and the KIT‐V559D and KIT‐K642E mutations, nintedanib displayed high potency in a dose‐dependent manner (Fig. 2B, Table S4).

To further ensure on‐target efficacy in GIST cell lines, we tested the response of the KIT‐mediated signalling pathway upon nintedanib treatment (Fig. 2C). Nintedanib potently inhibited the phosphorylation of KIT, such as Y703, Y719 and Y823, and downstream mediators, such as AKT and STAT3, which was also observed in cell lines with high expression RTK drug targets [35], in GIST‐T1 and GIST‐882 (imatinib sensitive) cells. Interestingly, in GIST‐5R (imatinib resistant) cells, nintedanib was more potent than sunitinib against KIT Y719 phosphorylation, which regulates SCF‐mediated cell migration [36], and both nintedanib and sunitinib displayed profound inhibitory effects on KIT Y703 and Y823 phosphorylation. As expected, in the GIST‐48B cell line, which is KIT independent, neither compound showed an effect on the downstream mediators of the KIT signalling pathway.

We then evaluated the effects of nintedanib on cell cycle progression. The results showed that nintedanib induced cell cycle arrest in the G_0_‐G_1_ phase starting at 30 nm in GIST‐T1 and GIST‐882 (imatinib sensitive) and GIST‐5R (imatinib resistant) cells but not in GIST‐48B (KIT independent) cells (Fig. 2D). Nintedanib induced the apoptosis of KIT‐mutant GIST cell lines but not the KIT‐independent GIST‐48B cell line (Fig. 2E). Together, these data demonstrated that nintedanib suppressed proliferation and KIT pathway signalling in imatinib‐resistant cells harbouring a KIT‐dependent resistance mutation and preclinical GIST models *in vitro*.

### 3.3 *In vivo* antitumour efficacy of nintedanib against GIST‐T1, KIT‐T670I/BaF3, GIST‐T1‐T670I and GIST‐5R mouse xenograft models

**Fig. 3:**
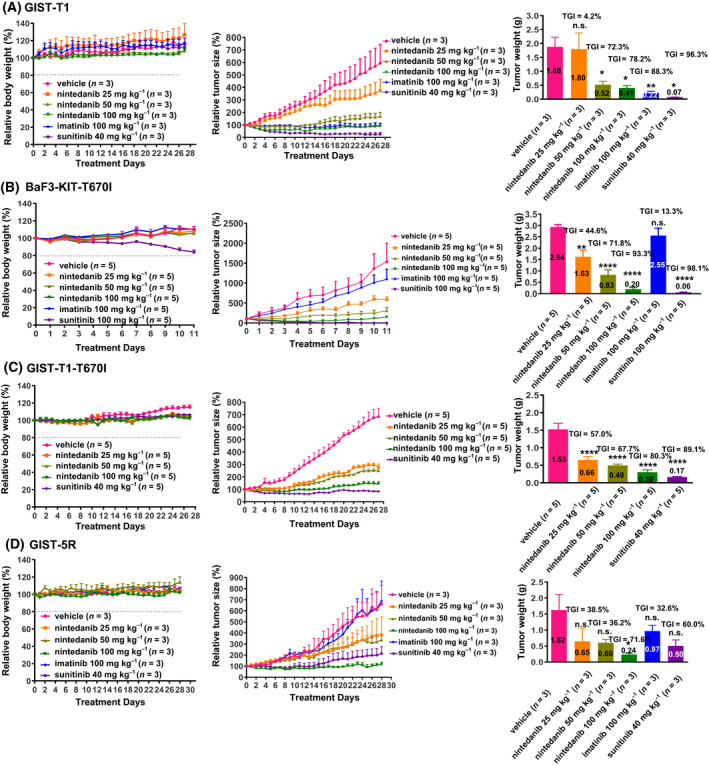
*In vivo* antitumour efficacy of Nintedanib against GIST‐T1, KIT‐T670I/BaF3, GIST‐T1‐T670I and GIST‐5R mouse xenograft model. (A) Effects of nintedanib on GIST‐T1 mouse xenograft model (*n* = 3, independent experiments). (B) Effects of nintedanib on BaF3‐KIT‐T670I mouse allograft model (*n* = 5, independent experiments). (C) Effects of nintedanib on GIST‐T1‐T670I mouse xenograft model (*n* = 5, independent experiments). (D) Effects of nintedanib on GIST‐5R mouse xenograft model (*n* = 3, independent experiments). Animals were treated orally once a day with a various of dose of the drugs. Data are shown as mean ± SEM, n.s., not significant; *P‐value < 0.05; **P‐value < 0.01; ****P < 0.0001 (one‐way ANOVA).

GIST‐T1, KIT‐T670I/BaF3, GIST‐T1‐T670I and GIST‐5R mouse xenograft models were used to assess the *in vivo* antitumour efficacy of nintedanib. At doses as high as 100 mg·kg^−1^·day^−1^, nintedanib caused no significant body weight changes in any treatment group. In the GIST‐T1 mouse xenograft models, tumour growth was suppressed by nintedanib in a dose‐dependent manner, and the tumour growth inhibition (TGI) rate was 72.3% at the 50 mg·kg^−1^ dose and 78.2% at the 100 mg·kg^−1^ dose (Fig. 3A). In the KIT‐T670I/BaF3‐ and GIST‐T1‐T670I‐inoculated mouse models, 100 mg·kg^−1^ nintedanib nearly completely suppressed tumour growth, with TGI values of 93.3% and 80.3%, respectively, while imatinib showed a limited effect on tumour growth at the same doses (Fig. 3B,C). As expected, nintedanib inhibited the KIT signalling pathway in KIT‐T670I/BaF3 tumour tissues in a dose‐dependent manner (Fig. S2). In the GIST‐5R mouse xenograft models, tumour growth was inhibited by nintedanib in a dose‐dependent manner, and the TGI rate was 71.6% at 100 mg·kg^−1^, while imatinib had no effect on tumour growth (Fig. 3D). Immunohistochemical (IHC) staining showed that nintedanib inhibited cell proliferation and induced cell apoptosis in the GIST‐T1 and GIST‐5R mouse xenograft models in a dose‐dependent manner (Figs S3 and S4). These results confirmed that nintedanib elicited robust antitumour efficacy in KIT Δ560–578 and T670I mutation‐dependent tumour models at well‐tolerated doses.

### 3.4 Nintedanib inhibits the proliferation and KIT signalling pathway in human primary GIST cells in the presence of FGF2 ligand

**Fig. 4:**
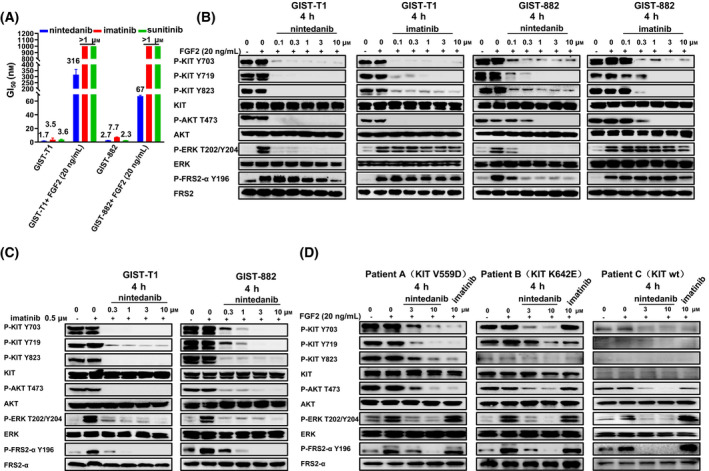
Anti‐proliferation of human GIST cancer cell lines and patient‐derived primary cells after treating with nintedanib and imatinib within FGF‐2. (A) Effects of nintedanib, imatinib and sunitinib on proliferation of GIST‐T1 and GIST‐882 cell lines within 20 ng·mL^−1^ FGF2 for 3 days using CellTiter‐Glo assay. Data are shown as mean ± SD (*n* = 3, independent experiments). (B) Effects of nintedanib and imatinib on the FGFR and KIT signalling pathways in GIST‐T1 and GIST‐882 cell lines within 20 ng·mL^−1^ FGF2 for 4 h (immunoblotting; *n* = 3, independent experiments). (C) GIST‐T1 and GIST‐882 cell lines were treated with imatinib for 4 h to activate FGF/FGFR signalling pathway, after which imatinib was removed and the cells were treated with nintedanib for 4 h for immunoblotting analysis. This experiment was conducted once. (D) Effects of nintedanib and imatinib on the FGFR and KIT signalling pathways in three GIST patients within 20 ng·mL^−1^ FGF2 for 4 h (immunoblotting).

As previously reported [26], imatinib resistance in GISTs in which FGF2 is universally highly expressed can be induced by MAP kinase activation through FGF signalling. Therefore, we next investigated whether FGFR‐mediated reactivation of the MAPK pathway can attenuate the antiproliferative effect of nintedanib in GISTs (Fig. 4A). The results showed that nintedanib was more potent than imatinib and sunitinib against the GIST‐T1 and GIST‐882 cells with FGF signalling pathway activated by FGF2 ligand. To address the molecular mechanism underlying the growth inhibition of the GIST‐T1 and GIST882 cell line by nintedanib in the presence of 20 ng·mL^−1^ FGF2, we tested the activated states of downstream mediators of the KIT and FGF signalling pathways (Fig. 4B). As expected, nintedanib strongly inhibited the KIT downstream signalling pathway and phospho‐FRS2α, the major downstream substrate of FGFRs. By comparison, imatinib inhibited only the autophosphorylated KIT and downstream signalling pathways but not ERK, which was due to reactivated FGFR signalling. To test whether nintedanib can overcome the resistance induced by imatinib, which enables FGFR signalling activation, we examined the effects of nintedanib on KIT and FGFR‐mediated signalling pathways after imatinib treatment (Fig. 4C). The results demonstrated that nintedanib could strongly inhibit FGFR signalling. Interestingly, the phosphorylation of histone 2A family member X (γ‐H2AX) [37], which is an indication for DNA injury, was remarkably increased in this experiment (Fig. S5). We then tested the effects of nintedanib on human primary GIST cells that expressed the KIT‐V559D, KIT‐K642E and KIT‐wt in KIT (Fig. 4D). The results showed that nintedanib could strongly inhibit the autophosphorylated KIT, phospho‐FRS2α and KIT downstream signalling pathways with 20 ng·mL^−1^ FGF2. In addition, nintedanib increased the phosphorylation of H2AX in the primary tumour cells derived from KIT‐V559D and KIT‐K642E GIST patients, which suggested that nintedanib could induce apoptosis in part through DNA double‐strand breaks (Fig. S6).

### 3.5 Nintedanib inhibits tumour growth of a GIST882 mouse xenograft model *in vivo* without activation of the FGF signalling pathway

**Fig. 5:**
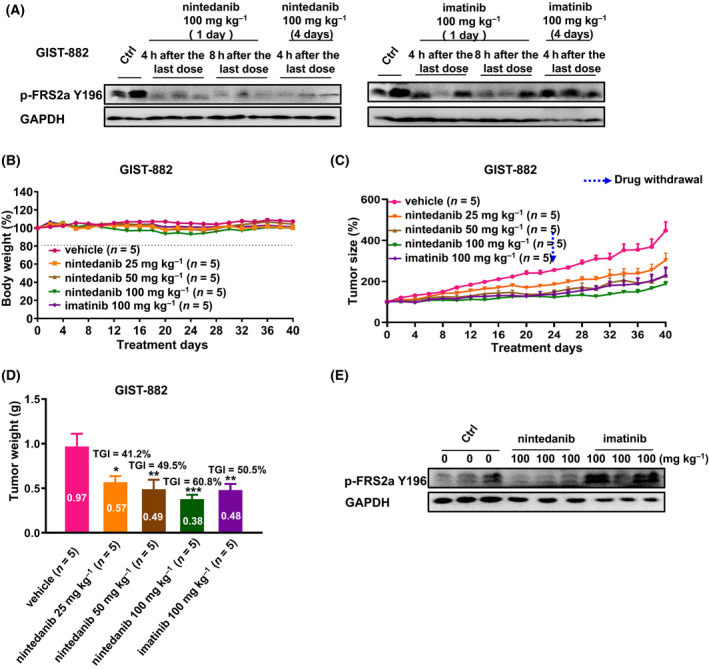
Effect of Nintedanib and Imatinib in GIST‐882 mouse xenograft models *in vivo*. (A) Effects of nintedanib and Imatinib on the FGFR signalling pathways in GIST‐882 mouse xenograft models (control, *n* = 2, independent experiments; treated, *n* = 3, independent experiments). (B‐D) Effect of nintedanib and imatinib on GIST‐882 mouse xenograft models (*n* = 5, independent experiments). (E) Effects of nintedanib and imatinib on the FGFR signalling pathways in GIST‐882 mouse xenograft models after treated by 40 days (*n* = 3, independent experiments). Animals were treated orally once a day with a various of dose of the drugs. Data are shown as mean ± SEM, *P‐value < 0.05; **P‐value < 0.01; ***P‐value < 0.001 (one‐way ANOVA).

Considering a previous report showing that the effect of imatinib was reduced in a GIST882 xenograft model by inducing activation of FGFR signalling [26], we tested the antitumour efficacy of nintedanib on GIST882 tumour‐bearing mice. A GIST882 xenograft model was treated with imatinib or nintedanib twice daily for 1 day and 4 days. The phosphorylation of FRS2α was increased after treatment with imatinib for 4 days, a finding not found with nintedanib treatment and was consistent with the imatinib‐induced activation of the FGF signalling pathway (Fig. 5A). We next determined whether nintedanib was more effective than imatinib *in vivo*. In the GIST882 cell‐inoculated mouse xenograft model, nintedanib at doses as high as 100 mg·kg^−1^·day^−1^ showed no apparent toxicity (Fig. 5B). Tumour growth was inhibited by nintedanib in a dose‐dependent manner, and the TGI rate was 60.8% at 100 mg·kg^−1^·day^−1^, whereas imatinib showed a limited effect on tumour growth at the same dose. After drug withdrawal, nintedanib displayed a long‐lasting response (Fig. 5C,D). As expected, nintedanib, but not imatinib, reduced the phosphorylation of FRS2α, which is a downstream substrate of FGFR (Fig. 5E). These results support the notion that the application of nintedanib may be a new strategy for enhancing the treatment for GIST patients with *de novo* or acquired resistance to imatinib.

## 4 Discussion

Because of advancements in next‐generation sequencing technology, it has been found that secondary mutations of kinases are not the only mechanisms of drug resistance and that the activation of alternative pathways can also contribute to drug resistance. For instance, imatinib, as a first‐line therapy, has significantly improved GIST patient survival [38]. However, most patients eventually experience disease progression due to KIT secondary mutations, including activation loop and gatekeeper mutations [39, 40]. Although regorafenib, sunitinib and ripretinib have been approved to overcome imatinib‐resistant mutations of KIT, they both show toxicity and poor clinical responses [41]. Several new KIT inhibitors against imatinib‐resistant secondary mutations are currently being evaluated in clinical trials, such as pazopanib [42], anlotinib [43] and dovitinib [44]. In addition, many candidate KIT inhibitors are in preclinical development, such as CHMFL‐KIT‐033/8140 [31, 45] and AZD3229 [46]. However, it takes a long time to complete clinical research, and these drugs are temporarily unable to fulfil the urgent clinical demand.

It has been demonstrated that imatinib resistance in GISTs is caused by the feedback activation of FGF signalling, which results in a rebound in ERK phosphorylation [26, 47]. The combination of the pan‐FGFR inhibitors BGJ398 and imatinib repressed ERK rebound and enhanced the antitumour activity of imatinib in GISTs. In addition, the Memorial Sloan Kettering Cancer Center (MSKCC) conducted a phase I clinical trial with a combination of imatinib and BGJ398 (Trial Registration: NCT02257541) for patients with locally advanced or metastatic GISTs. Approximately 30% of the evaluable study cohort attained durable stabilization of disease lasting at least 32 weeks. However, combination toxicity was an important factor that limited the ability of this study to meet its primary end point [48]. Therefore, there remains an urgent need for more agents that can inhibit both the different resistance mutations of KIT and FGFRs, which are usually highly expressed in GISTs and participate signalling crosstalk with KIT, causing imatinib resistance [49].

In this study, through high‐throughput screening using a functional isogenic BaF3 cell library, we found that nintedanib, a triple angiokinase inhibitor of FGFR/VEGFR/PDGFR that has been approved for the treatment of IPF and NSCLC, exhibited potent activity against a panel of primary gain‐of‐function (GOF) mutations and secondary drug resistance mutations in KIT kinase. Interestingly, in addition to better efficacy against KIT wt, nintedanib showed potent inhibitory effects against primary GOF mutations (V559A/D/G and L576P), a few secondary imatinib‐resistant mutations in the ATP binding pocket (such as V654A and T670I) and activation loops (such as D820E/G/Y, D816E/H/V, A829P) and sunitinib‐resistant mutations such as D816H/V and A829P). Because nintedanib is a multi‐targeted kinase inhibitor, we believe that this antitumour activity is not only contributed by KIT inhibition alone, its inhibition on other kinases, such as VEGFR and PDGFR, may also play a role. However, nintedanib could not completely overcome the resistance mediated by MAP kinase activation via FGF signalling, which may be due to the low drug concentration in tumours or the weak inhibition of FGFR by nintedanib.

## 5 Conclusions

In summary, nintedanib eliminated the adaptive response after KIT inhibition in imatinib‐sensitive GISTs. Additionally, nintedanib may be effective in the imatinib‐resistant clones containing acquired mutations in KIT kinase and FGFR feedback activation that have emerged. The findings described here support a basis for extending the application of nintedanib as a new strategy to improve the treatment of GIST patients with *de novo* or acquired resistance to imatinib.

## Conflict of interest

The authors declare no conflict of interest.

## Author contributions

JiL and QL supervised and conceptualized the study. JiL and QL wrote the manuscript. JLu, JG, AW and ZJ performed most of the experiments. SQ, ZQ, FL, KY, JC, CC, CH, HW, LW and WW helped with project design and provided guidance or materials for some experiments. All authors have read and approved the final manuscript.

### Peer review

The peer review history for this article is available at https://publons.com/publon/10.1002/1878‐0261.13199.

## Supporting information


**Fig. S1**. The phosphorylation levels of KIT Y703, Y719, and Y823 were detected by western blot in a panel of KIT kinase transformed isogenic BaF3 cell lines.
**Fig. S2**. Effect of nintedanib, imatinib, and sunitinib on the KIT‐mediated signaling pathways in KIT‐T670I/BaF3 xenograft mouse models.
**Fig. S3**. Immunohistochemistry staining of the tumor tissues with nintedanib treatment.
**Fig. S4**. The percentage of Ki67 and TUNEL positive cells was also calculated and shown as graphs.
**Fig. S5**. The phosphorylation levels of H2AX‐S139 were detected by western blot in GIST‐T1 and GIST‐882 cell lines.
**Fig. S6**. The phosphorylation levels of H2AX‐S139 were detected by western blot in 2 GIST patients.
**Table S1**. Anti‐proliferative effect of nintedanib, imatinib, sunitinib, avapritinib, and ripretinib against a panel of Ba/F3 isogenic cell lines.
**Table S2**. Inhibitory activity of nintedanib, imatinib, and sunitinib to the phosphorylation of KIT Y703/ Y719/ Y823 in a panel of BaF3 cells.
**Table S3**. ADP‐Glo™ assay determination of the IC_50_ values of nintedanib against KIT WT and mutant proteins.
**Table S4**. Clinical data of patient primary cells.

## Data Availability

The data presented in this study are available on request from the corresponding author.
