# Supplementary material for: Nintedanib overcomes drug resistance from upregulation of FGFR signalling and imatinib‐induced KIT mutations in gastrointestinal stromal tumours
Source: Mol Oncol. 2022 Mar 6;16(8):1761–74. doi: 10.1002/1878-0261.13199 (PMC9019892; doi:10.1002/1878-0261.13199)
Supplement: Supplementary file 1 — Fig. S1. The phosphorylation levels of KIT Y703, Y719, and Y823 were detected by western blot in a panel of KIT kinase transformed isogenic BaF3 cell lines. Fig. S2. Effect of nintedanib, imatinib, and sunitinib on the KIT‐mediated signaling pathways in KIT‐T670I/BaF3 xenograft mouse models. Fig. S3. Immunohistochemistry staining of the tumor tissues with nintedanib treatment. Fig. S4. The percentage of Ki67 and TUNEL positive cells was also calculated and shown as graphs. Fig. S5. The phosphorylation levels of H2AX‐S139 were detected by western blot in GIST‐T1 and GIST‐882 cell lines. Fig. S6. The phosphorylation levels of H2AX‐S139 were detected by western blot in 2 GIST patients. Table S1. Anti‐proliferative effect of nintedanib, imatinib, sunitinib, avapritinib, and ripretinib against a panel of Ba/F3 isogenic cell lines. Table S2. Inhibitory activity of nintedanib, imatinib, and sunitinib to the phosphorylation of KIT Y703/ Y719/ Y823 in a panel of BaF3 cells. Table S3. ADP‐Glo™ assay determination of the IC50 values of nintedanib against KIT WT and mutant proteins. Table S4. Clinical data of patient primary cells. [file MOL2-16-1761-s001.docx]

**Supplemental Tables and Figures**

**Table S1:** Anti-proliferative effect of nintedanib, imatinib, sunitinib, avapritinib, and ripretinib against a panel of Ba/F3 isogenic cell lines^a^. The cells were treated with nintedanib, imatinib, sunitinib, avapritinib, and ripretinib (0-10 μM) for 72 h, and then cell viability was measured using the CellTiter–Glo assay.

| **Cell line (GI_50:_ μM)** | **Mutation** | **nintedanib** | **imatinib** | **sunitinib** | **avapritinib** | **ripretinib** |
| --- | --- | --- | --- | --- | --- | --- |
| **BaF3-tel-KIT** | / | 0.058 | 0.59 | 0.11 | 0.040 | 0.0035 |
| **BaF3-tel-KIT-L576P** | **Exon 11** | 0.0011 | 0.046 | 0.0063 | 0.128 | 0.0088 |
| **BaF3-tel-KIT-V559D** |  | <0.0003 | 0.0031 | 0.0031 | 0.045 | 0.001 |
| **BaF3-tel-KIT-V559G** |  | 0.0038 | 0.023 | 0.0011 | 0.040 | 0.001 |
| **BaF3-tel-KIT-V654A** | **Exon 13** | 0.036 | 1.4 | 0.001 | 0.523 | 0.031 |
| **BaF3-tel-KIT-T670I** | **Exon 14** | <0.0003 | 9.6 | 0.005 | 0.039 | 0.017 |
| **BaF3-tel-KIT-T670E** |  | 0.0025 | 3.53 | <0.0003 | 0.258 | 0.100 |
| **BaF3-tel-KIT-D816V** | **Exon 17** | 0.12 | 6.6 | 0.61 | 0.034 | 0.068 |
| **BaF3-tel-KIT-D816H** |  | 0.32 | ~10 | 1.0 | 0.094 | 0.028 |
| **BaF3-tel-KIT-D816E** |  | 0.044 | 0.12 | 0.01 | 0.087 | 0.0070 |
| **BaF3-tel-KIT-D820E** |  | 0.0012 | 0.12 | 0.011 | 0.046 | 0.0029 |
| **BaF3-tel-KIT-D820G** |  | 0.036 | 0.52 | 0.18 | 0.061 | 0.004 |
| **BaF3-tel-KIT-D820Y** |  | 0.0071 | 0.46 | 0.048 | 0.030 | 0.0015 |
| **BaF3-tel-KIT-Y823D** |  | 0.006 | 0.29 | 0.04 | 0.017 | 0.0005 |
| **BaF3-tel-KIT-A829P** | **Exon 18** | 0.06 | 0.48 | 0.12 | 0.027 | 0.0012 |
| **BaF3-tel-KIT-V654A-V559D** | **Exon 11,**  **13** | 0.093 | 1.0 | 0.0017 | 0.790 | 0.048 |
| **BaF3-tel-KIT-V559D-T670I** | **Exon 11,**  **14** | 0.0017 | >10 | 0.0016 | 0.498 | 0.029 |
| **BaF3** | / | 1.3 | >10 | 1.6 | 0.714 | 1.571 |

^a^ All GI_50_ values were obtained by triplet testing.

**Table S2** Inhibitory activity of nintedanib, imatinib, and sunitinib to the phosphorylation of KIT Y703/ Y719/ Y823 in a panel of BaF3 cells. The cells were treated with nintedanib, imatinib, and sunitinib (0-1000 nM) for 4 h, and then the cells lysed for western blot.

| **nintedanib (EC_50_: nM)** | **P-KIT Y703** | **P-KIT Y719** | **P-KIT Y823** |
| --- | --- | --- | --- |
| **BaF3-tel-KIT** | 4 | 54 | 89 |
| **BaF3-tel-KIT-V559D** | 46 | 72 | 21 |
| **BaF3-tel-KIT-V559G** | 5 | 7 | 4 |
| **BaF3-tel-KIT-L576P** | 12 | 33 | 53 |
| **BaF3-tel-KIT-T670E** | 61 | 147 | 34 |
| **BaF3-tel-KIT-T670I** | 5 | 25 | 4 |
| **BaF3-tel-KIT-D816E** | 67 | 179 | 64 |
| **BaF3-tel-KIT-D816H** | 107 | 324 | 41 |
| **BaF3-tel-KIT-D820E** | 9 | 46 | 51 |
| **BaF3-tel-KIT-Y823D** | 15 | 25 | 9 |
| **BaF3-tel-KIT-A829P** | 126 | 49 | 184 |
| **BaF3-tel-KIT-V654A-V559D** | 132 | 26 | 129 |
| **BaF3-tel-KIT-V559D-T670I** | 5 | 7 | 4 |

**Table S3:** ADP-Glo^TM^ assay determination of the IC_50_ values of nintedanib against KIT WT and mutant proteins. The biochemical assay was tested by Invitrogen (Carlsbad, CA, USA).

| **Target** | **nintedanib (nM)** |
| --- | --- |
| **KIT** | 3.85±0.84 |
| **KIT V654A** | 7.94±0.66 |
| **KIT T670E** | 59.4±17.7 |
| **KIT T670I** | 1.65±0.25 |
| **KIT V559D T670I** | <0.5±0.07 |
| **KIT D816H** | 1.14±0.04 |
| **KIT D816V** | 2.38±0.26 |
| **KIT D820E** | 3.14±0.33 |
| **KIT N822K** | 2.60±0.07 |
| **KIT Y823D** | 3.50±1.1 |
| **KIT A829P** | 2.64±0.27 |

**Table S4:** Clinical data of patient primary cells.

| **Patient** | **Sex** | **Age (years)** | **Origin** | **IHC analysis** | **Grade** | **Treatment status** |
| --- | --- | --- | --- | --- | --- | --- |
| **Patient A** | Male | 57 | Stomach | CD117+, DOG-1+, CD34+, S-100-, Desmin-, Ki67 8% | G2 | Not treated |
| **Patient B** | Female | 62 | Stomach | CD117+, DOG-1+, CD34+, S-100-, Desmin-, Ki67 1% | G3 | Not treated |
| **Patient C** | Male | 55 | Small intestine | CD117+, DOG-1+, CD34+, S-100-, Desmin-, Ki67 30% | G3 | imatinib |


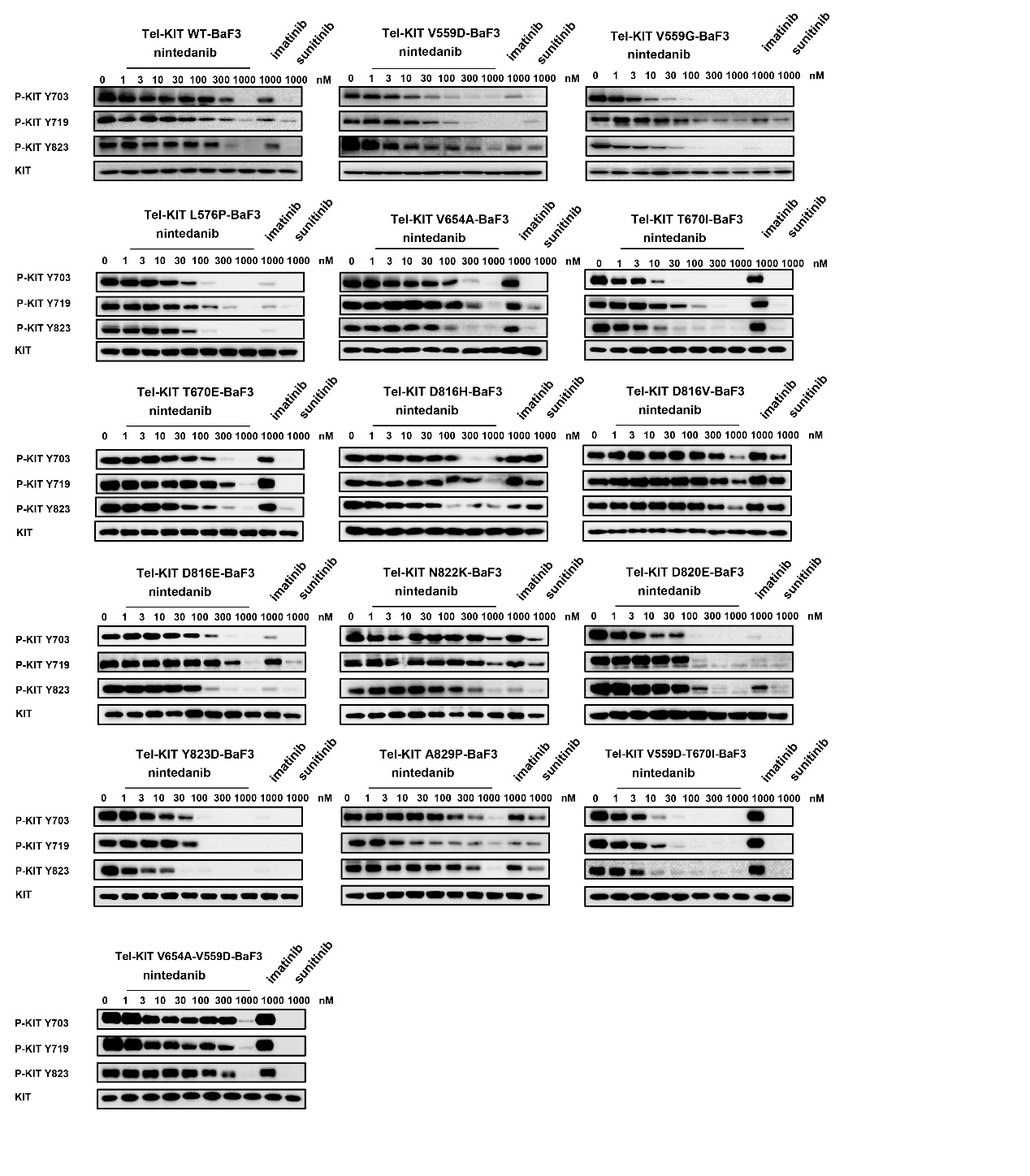


**Fig. S1**

The phosphorylation levels of KIT Y703, Y719, and Y823 were detected by western blot in a panel of KIT kinase transformed isogenic BaF3 cell lines. These cells were incubated with the indicated concentrations of nintedanib for 2 h before lysis. This experiment was conducted once.

**
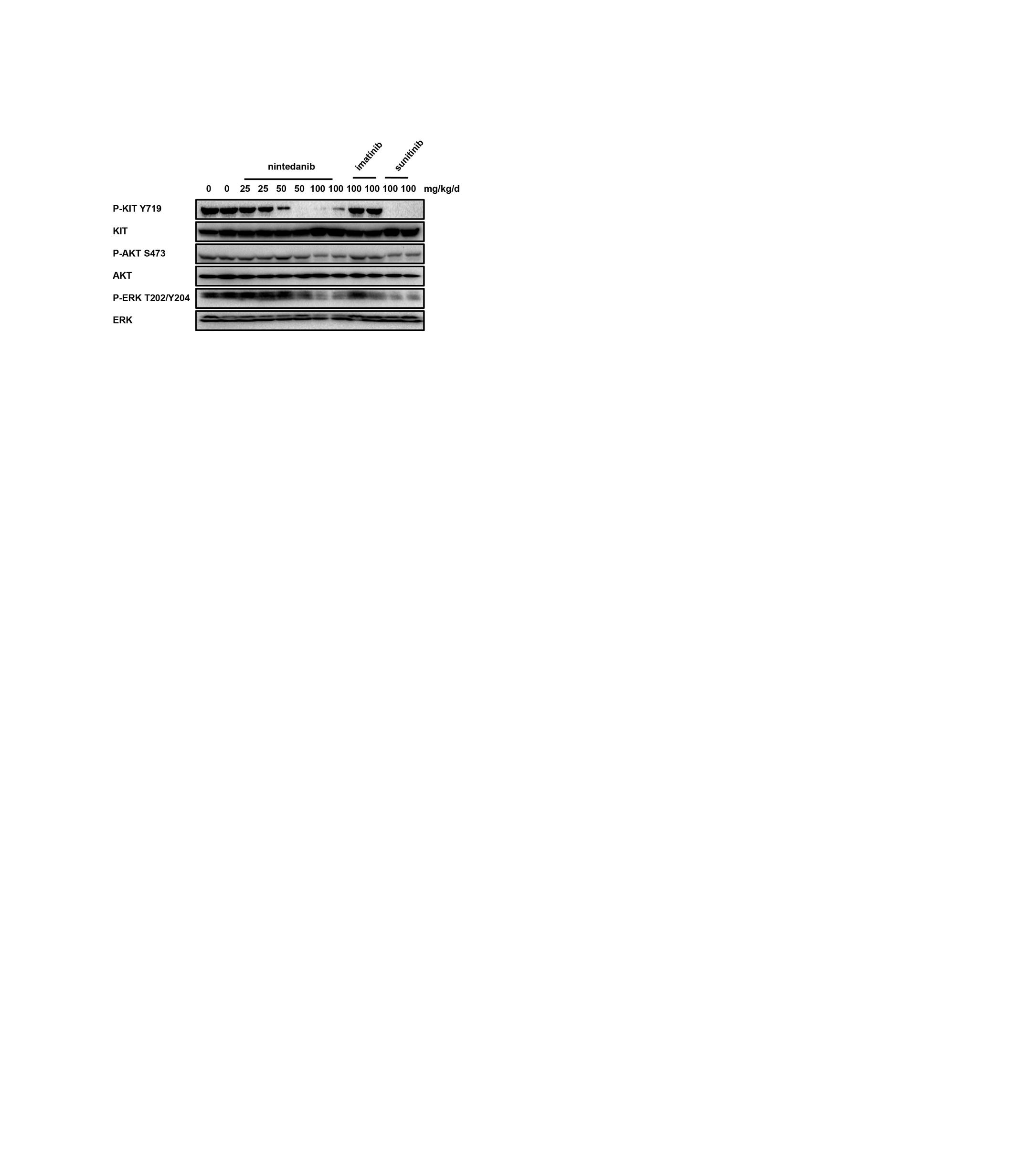
**

**Fig. S2** Effect of nintedanib, imatinib, and sunitinib on the KIT-mediated signaling pathways in KIT-T670I/BaF3 xenograft mouse models after 11-day treatment period (*n* = 2, independent experiments).


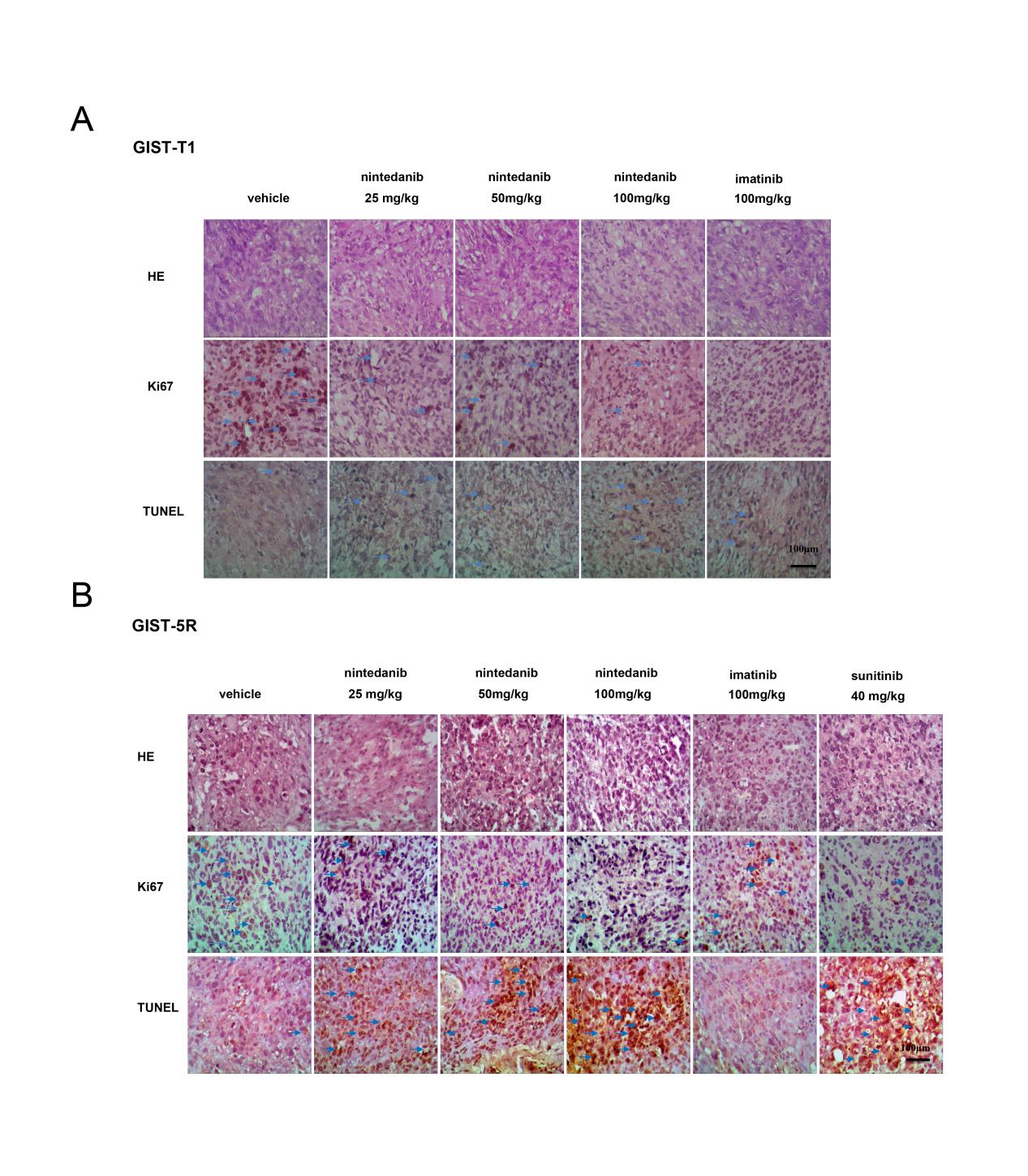


**Fig. S3** Immunohistochemistry staining of the tumor tissues with nintedanib treatment. (**A)** GIST-T1 xenograft model (*n* = 2, independent experiments); (**B)** GIST-5R xenograft model. Blue arrowheads, positive signals (*n* = 2, independent experiments).


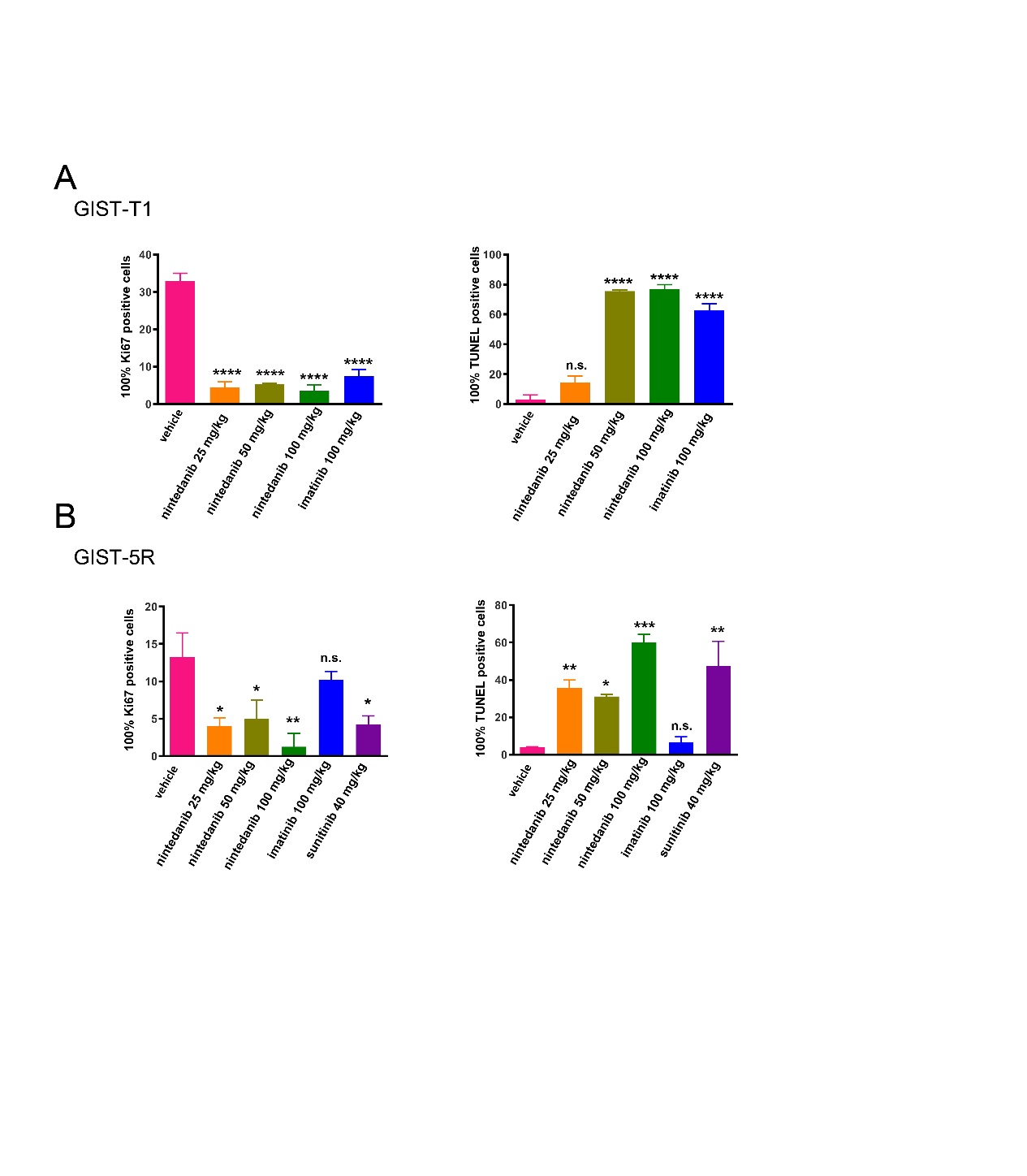


**Fig. S4** The percentage of Ki67 and TUNEL positive cells was also calculated and shown as graphs. (**A)** GIST-T1 xenograft model (*n* = 2, independent experiments); (**B)** GIST-5R xenograft model (*n* = 2, independent experiments). Data are shown as mean ± SD, n.s. = not significant; *P-value < 0.05; **P-value < 0.01; ***P-value < 0.001; ****p < 0.0001 (one-way ANOVA).


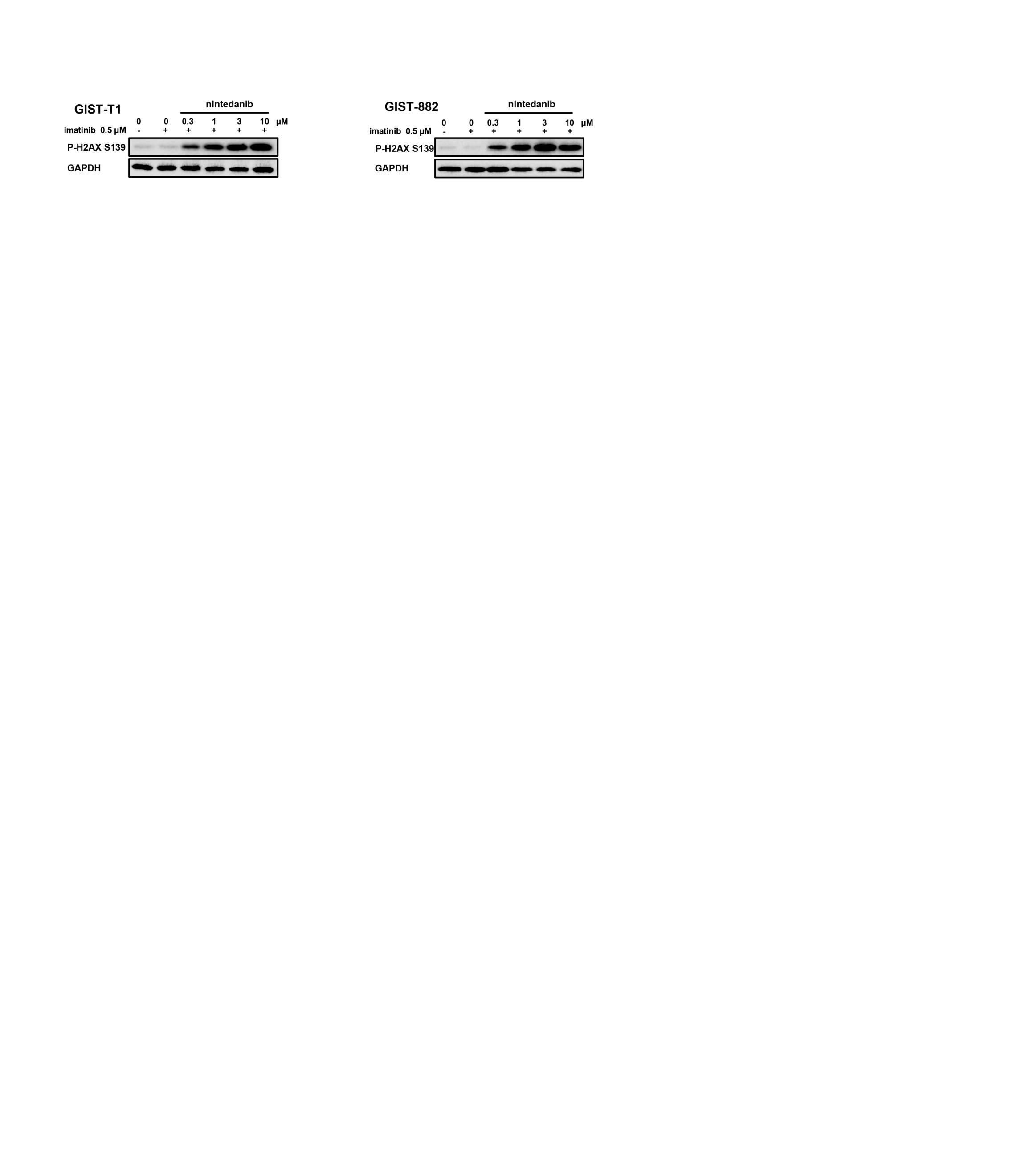


**Fig. S5** The phosphorylation levels of H2AX-S139 were detected by western blot in GIST-T1 and GIST-882 cell lines. These cells were incubated with the indicated concentrations of nintedanib for 4 h after 0.5 μM imatinib treatment before lysis. This experiment was conducted once.


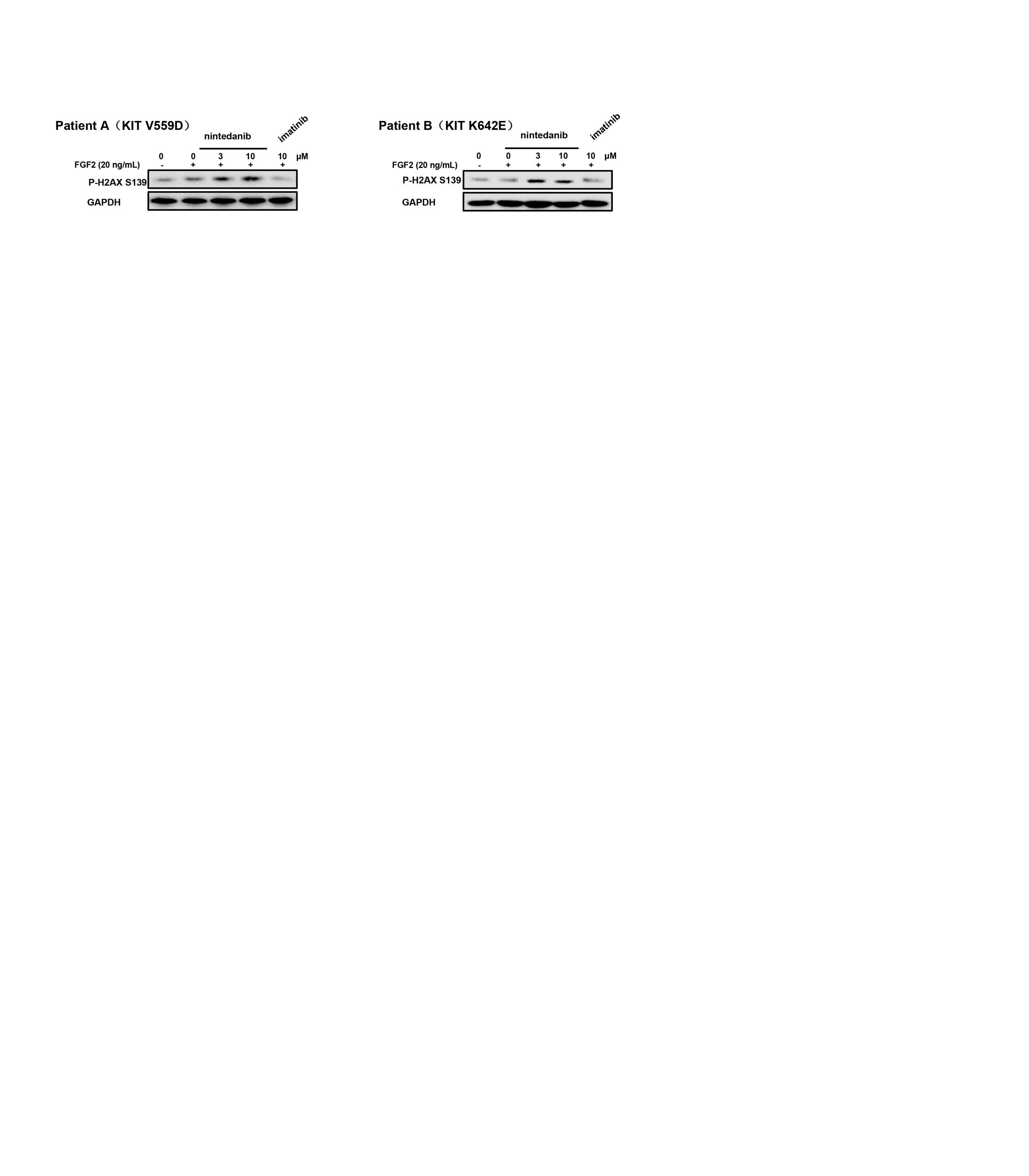


**Fig. S6** The phosphorylation levels of H2AX-S139 were detected by western blot in 2 GIST patients. These cells were incubated with the indicated concentrations of nintedanib for 4 h in the presence of 20 ng/mL FGF2 before lysis. This experiment was conducted once.
